# Safely reducing unnecessary benign breast biopsies by applying non-mass and DWI directional variance filters to ADC thresholding

**DOI:** 10.1186/s12880-022-00897-0

**Published:** 2022-09-29

**Authors:** Alan Penn, Milica Medved, Hiroyuki Abe, Vandana Dialani, Gregory S. Karczmar, David Brousseau

**Affiliations:** 1 Alan Penn and Associates, Inc., Rockville, MD 20850 USA; 2 University of Chicago, Chicago, IL 60637 USA; 3 Beth Israel Deaconess Medical Center, Boston, MA 02467 USA; 4 Mercy Medical Center, Merced, CA 95340 USA

**Keywords:** Breast DWI, Avoidable biopsies, ADC threshold, Non-mass filter, Directional variance filter

## Abstract

**Background:**

Thresholding apparent diffusion coefficient (ADC) maps obtained from Diffusion-Weighted-Imaging (DWI) has been proposed for identifying benign lesions that can safely avoid biopsy. The presence of malignancies with high ADC values leads to high thresholds, limiting numbers of avoidable biopsies.

**Purpose:**

We evaluate two previously reported methods for identifying avoidable biopsies: using case-set dependent ADC thresholds that assure 100% sensitivity and using negative likelihood ratio (LR-) with a fixed ADC threshold of 1.50 × 10^–3^ mm^2^/s. We evaluated improvements in efficacy obtained by excluding non-mass lesions and lesions with anisotropic intra-lesion morphologic characteristics.

**Study type:**

Prospective.

**Population:**

55 adult females with dense breasts with 69 BI-RADS 4 or 5 lesions (38 malignant, 31 benign) identified on ultrasound and mammography and imaged with MRI prior to biopsy.

**Field strength/sequence:**

1.5 T and 3.0 T. DWI.

**Assessment:**

Analysis of DWI, including directional images was done on an ROI basis. ROIs were drawn on DWI images acquired prior to biopsy, referencing all available images including DCE, and mean ADC was measured. Anisotropy was quantified via variation in ADC values in the lesion core across directional DWI images.

**Statistical tests:**

Improvement in specificity at 100% sensitivity was evaluated with exact McNemar test with 1-sided *p*-value < 0.05 indicating statistical significance.

**Results:**

Using ADC thresholding that assures 100% sensitivity, non-mass and directional variance filtering improved the percent of avoidable biopsies to 42% from baseline of 10% achieved with ADC thresholding alone. Using LR-, filtering improved outcome to 0.06 from baseline 0.25 with ADC thresholding alone. ADC thresholding showed a lower percentage of avoidable biopsies in our cohort than reported in prior studies. When ADC thresholding was supplemented with filtering, the percentage of avoidable biopsies exceeded those of prior studies.

**Data conclusion:**

Supplementing ADC thresholding with filters excluding non-mass lesions and lesions with anisotropic characteristics on DWI can result in an increased number of avoidable biopsies.

## Introduction

MR Diffusion Weighted Imaging (DWI) is an unenhanced procedure which can be used in conjunction with MR Dynamic Contrast Enhanced Imaging (DCE-MRI) to increase specificity and prevent unnecessary breast biopsies [1–4]. Breast lesions identified on DWI can be classified using Apparent Diffusion Coefficient (ADC) values by setting an ADC cutoff threshold equal to the maximum ADC value of malignant lesions (ADC_MaxMalig_). Benign lesions with ADC values > ADC_MaxMalig_ are additional lesions that could potentially avoid biopsy without missing cancers. By definition, using ADC_MaxMalig_ as a threshold results in 100% sensitivity, since all malignant lesions have ADC values ≤ ADC_MaxMalig_. We refer to this as the “100%-sensitivity method.” The performance measure of the100%-sensitivity method is the percentage of benign lesions that can potentially avoid biopsy without missing a cancer.

ADC values can vary significantly from one facility to another depending upon several factors including the patient population and the ROI drawing method [3, 5], and the value of ADC_MaxMalig_ will vary with the dataset being analyzed. Partridge, et al. reported ADC_MaxMalig_ = 1.81 × 10^–3^ mm^2^/s evaluating a dataset with 31 BI-RADS category 4 and 5 lesions [4]. Rahbar, et al., reported ADC_MaxMalig_ = 1.53 × 10^–3^ mm^2^/s evaluating data from the A6702 Trial which included 28 BI-RADS category 4 and 5 malignant lesions [6]. When the 100% sensitivity method is applied to a dataset with a small number of malignant lesions, there may be few, or possibly no, malignant lesions with very high ADC values (e.g., > 1.7 × 10^–3^ mm^2^/s), as was the case in Rahbar, et al.

Whereas the 100%-sensitivity method results in all malignant lesions being classified correctly, a second, less strict, criterion allows for a minimal number of cancers to be misclassified as benign. This second classification, based on BI-RADS categories, also uses an ADC threshold which is then used to derive the likelihood of malignancy. If an additional test reduces the post-test probability of cancer to ≤ 2%, category 4 lesions can potentially be reclassified to category 3, reducing the number of unnecessary biopsies [7, 8]. The Negative Likelihood Ratio, (LR-) is the performance measure of this evaluation method. Researchers have used LR- = 0.1 as the upper limit for acceptable downgrading from BI-RADS Category 4 to Category 3 [3, 8]. LR- is a function of the ADC threshold that is used. Clauser, et al. used an ADC threshold of 1.5 × 10^–3^ mm^2^/s, based on ADC_MaxMalig_ = 1.53 × 10^–3^ mm^2^/s in Rahbar, et al. [3, 6]. Our analysis follows Clauser, et al. and similarly uses an ADC threshold of 1.5 × 10^–3^ mm^2^/s. We refer to this as the likelihood-of-malignancy method or “LM method.”

The 100%-sensitivity method and the LM method showed effectiveness in the studies cited above, but showed minimal effectiveness when applied to the cases used in this study. This was due to our cases having a high ADC_MaxMalig_, excluding a substantial number of benign lesions from the set of potentially avoidable biopsies. To mitigate this limitation, we introduced two additional filters, non-mass and directional variance filters, which had been found to be effective on some malignant lesions with high ADC values. [9].

The objective of this study was to evaluate whether the proposed non-mass and directional variance filters improve the fraction of benign lesions that can safely avoid biopsy in a typical clinical situation. Both the 100%-sensitivity and the LM methods were analyzed.

## Materials and methods

### Patient recruitment and imaging protocol

The study was performed under an IRB-approved protocol, with informed consent obtained from all subjects. The patient ages ranged from 38 to 74 years old (median 56.5 years). Patients with breast lesions found on mammographic and/or sonographic exams for whom biopsy was recommended (BI-RADS 4 or BI-RADS 5) [10] were recruited prospectively before breast biopsy was performed. Subjects who had undergone prior treatment (e.g., chemotherapy, radiation therapy, excisional biopsy) that could distort diffusion signals were excluded. Fifty-five patients with 69 lesions (38 malignant; 31 benign) were imaged between Jan. 1, 2015 and Nov 15, 2016. Patients underwent MRI with DWI before biopsy. Lesion characteristics are given in Table 1. No lesions were excluded because of imaging problems or patient motion.

**Table 1:** Lesion characteristics

Characteristic	No. of Lesions	% ^§^
*Size (cm*^*2*^*)*		
< 1.0	29	42.0
1.0–2.0	17	24.6
> 2.0	23	33.3
*Type*		
Mass	51	73.9
Non-mass	18	26.1
*Mammographic density*		
Extreme	11	15.9
Heterogeneous	58	84.1
*Histology*		
Malignant (all)	38	55.1
IDC	15	21.7
IDC with DCIS	10	14.5
DCIS	7	10.1
ILC	5	7.2
Metaplastic carcinoma	1	1.4
Benign (all)	31	44.9
Fibroadenoma	11	15.9
Intraductal papilloma	4	5.8
Apocrine metaplasia	4	5.8
Adenosis	2	2.9
UDH	2	2.9
Stromal proliferation	1	1.4
Perilobular and periductal inflammation	1	1.4
Focal stromal fibrosis	1	1.4
Complex sclerosing lesion	1	1.4
PASH	1	1.4
Chronic inflammation	1	1.4
Atrophic changes	1	1.4
ADH	1	1.4

^§^ Percentages do not add to 100% due to rounding

“Extreme”—extremely dense breast tissue, “Heterogeneous” —heterogeneously dense breast tissue, “IDC”—invasive ductal carcinoma, “DCIS”—ductal carcinoma in situ, “ILC”—invasive lobular carcinoma, “UDH”—usual ductal hyperplasia, “PASH”—pseudoangiomatous stromal hyperplasia, “ADH”—atypical ductal hyperplasia

All subjects underwent DWI, non-fat suppressed T2-weighted imaging, and DCE-MRI using dedicated 16-channel Mammotrack phased array breast coils (Philips Healthcare, Best, Netherlands), at a 1.5 T Achieva (Philips Healthcare, Best, Netherlands; 2 benign; 3 malignant lesions) and a 3 T Achieva (Philips Healthcare, Best, Netherlands; 29 benign; 35 malignant lesions). Diffusion weighted images were acquired prior to the administration of gadolinium-based contrast agent and the acquisition of DCE-MRI. Spin-echo echo-planar imaging (SE-EPI) was used to generate diffusion weighted images and corresponding ADC maps in the axial plane. DWI data were acquired, retained, and analyzed individually for each of the three diffusion gradient encoding directions: phase (P), readout (R), and slice (S). Imaging parameters for the diffusion-weighted sequences are given in Table 2.

**Table 2:** Diffusion-weighted imaging parameters

	Philips Achieva 1.5 T	Philips Achieva 3.0 T
TR [ms]	16,860–16,960	10,546–13,863
TE [ms]	80.1	63.9–67.5
Field-of-view [mm^2^]	300 × 300–330 × 330	300 × 300–390 × 390
In-plane resolution [mm]	1.15–1.25	1.04–1.25
Slice thickness [mm]	2.5	2.5
Number of slices	80	65–80
b values [s/mm^2^]	0, 800	0, 800

“TR”—repetition time, “TE”—echo time

### ROI Definition

The radiologist whose annotations were used in this study (HA) is a fellowship-trained breast radiologist with over 10 years of experience who reads breast MRI as part of his clinical practice. The reader (HA), who was familiar with clinical results and had access to mammographic, sonographic and DCE-MRI images in addition to DWI images, selected one of the ADC, b = 0 s/mm^2^ or b = 800 s/mm^2^ series for lesion delineation based on assessment of lesion visibility. The reader selected the set of axial slices containing the lesion that would be annotated and drew lesion ROIs on each of the selected axial slices. Lesion size measurements in cm^2^ were recorded on the original annotated images for each annotated slice. For each case, the axial slice with the largest ROI was designated as the “index slice.”

When a lesion identified on mammography was non-enhancing at DCE-MRI, the DWI ROI was drawn by the reader (HA) on the ADC map by referencing the mammographic images for localization and determination of lesion extent. Filters are based upon non-mass characterization and identification of anisotropic morphologic features through analysis of directional intra-lesional diffusion DWI on a per-lesion basis.

### Mass/non-mass designation

All lesions were visually assessed as being masses or non-mass lesions based upon the principles of the BI-RADS DCE-MRI lexicon and assessment standards, [10] with non-mass enhancement (NME) type lesions designated “Non-mass” in this study. Two lesions which were seen on mammography were negative on DCE-MRI: one group of calcifications (benign: atrophic changes with ADH) and one asymmetry (benign: focal stromal fibrosis). For these lesions without enhancement, the visual assessment of mass/non-mass morphology was made by a second reader (DB) blinded to both pathology and ADC values after reviewing the mammography images. The morphologic designation was determined by substituting the shape of the ADC ROI drawn by the first reader in lieu of the shape referenced on the DCE-MRI image.

### Image scaling and ROI mapping from annotated images to DICOM-size images

DWI images were initially upsampled from the DICOM (“native”) resolution to a higher resolution review monitor (1680 × 1050) pixel HP Compaq LA2205wg monitor—Hewlett-Packard, Palo Alto, CA) to improve the visibly identifiable margins of any border selected by a radiologist when annotating an ROI around lesions. ROIs were drawn on upsampled images after image resolution had been increased from a native range of 240 × 240 to 336 × 336 pixels, to an upsampled range of 504 × 526 to 1274 × 994 pixels. The annotating system computed and displayed the size of the ROIs in cm^2^. For analysis, ROIs on native resolution images were constructed from the ROIs on upsampled images using the following procedure: First, the annotated image was cropped and downscaled to the dimensions, in pixels, to that of the native resolution image preserving, as much as possible, the size of the ROI in mm^2^. Second, the dilation operator was applied to the ROI in the native resolution image to add a 1-pixel wide band around the pixels in the constructed ROI; the 1-pixel wide expansion augmented signal captured from the lesion periphery and increased the number of sample points for statistical analysis.

The same ROI was used for all native resolution diffusion weighted images, including the three directional DWI images acquired with diffusion-encoding gradients applied in each of the three spatial directions.

### Application of secondary filtering to the process of lesion discrimination

To achieve improved discrimination of benign lesions in our data set while maintaining a 100% sensitivity threshold when using the 100%-sensitivity method and a high sensitivity when using the LM method, supplemental filters were applied sequentially to the data set. The methodology being assessed in this research discriminates lesion pathology based upon the following functions:

1. Filter 1: Non-mass lesions are identified morphologically and designated for biopsy.
2. Filter 2: All mass lesions are evaluated with quantitative assessment of directional DWI anisotropy (SDAC filtering) with high SDAC lesions designated for biopsy.
3. A mean lesion ADC cutoff threshold is then applied to all lesions to identify the final subset of lesions which may avoid unnecessary biopsy (i.e., the A6702 method).

### Filter 1: identification of non-mass lesions

Filter 1 forces all non-mass lesions into a suspicious classification by assigning an artificial ADC value of 0 to all non-masses (7 benign and 11 malignant non-masses). The rationale for applying this filter is discussed in the results and further elaborated upon in the discussion sections.

### Filter 2: quantification of lesion directional DWI anisotropy (i.e., SDAC filtering)

The SDAC feature is defined to be the standard deviation of area covered by pixels with ADC < 1.37 × 10^–3^ mm^2^/s, evaluated on augmented maps generated from DWI scans in the phase, readout, and slice directions. Augmented maps are generated independently for each of the three directional DWI acquisitions by multiplying ADC values by the corresponding signal intensities obtained at b = 0 s/mm^2^. Details of the SDAC feature and image augmentation methodology are given in [9] and described briefly below. Larger SDAC feature values, corresponding to increased anisotropy, are associated with a higher likelihood of malignancy.

To obtain SDAC values, for each axial slice, models of the lesion core are independently generated on augmented ADC maps for each of the three directional DWI scans, as follows. 3D “lesion models” are constructed by selecting an ADC threshold value and constructing a 3D volume of interest (VOI), extended over all slices containing the lesion that include voxels with ADC values lower than the threshold. These VOI are connected in 3D but not necessarily in the 2D index slice plane. The threshold value is varied to maximize the overlap of the radiologist-defined lesion ROI and the VOI cross-section in the index slice plane, and this overlap is defined as the “lesion core”. [9].

### Definition of filtered ADC values: “ADC-M” and “ADC-MD”

ADC-M (ADC with mass filter) assigns an ADC value of 0 to all non-masses which prevents non-masses from being classified as possibly benign. The ADC value obtained from the ROI of a mass is not altered by this filter.

ADC-MD (ADC with mass and directional filters) is defined as ADC-M for all lesions with SDAC < 4.5 mm^2^ and equal to 0 for lesions with SDAC > 4.5 mm^2^. The application of this second filter further restricts the set of possibly benign lesions with potentially avoidable biopsies to those with SDAC < 4.5 mm^2^. Maximum specificity at 100% sensitivity was achieved using SDAC threshold in the range [4.0 mm^2^–5.0 mm^2^]; a value of 4.5 mm^2^ was selected as representative of this range.

### Evaluation criteria

#### 100%-sensitivity evaluation

Following the methodology of A6702, ADC thresholds for 100% sensitivity are defined as ADC_MaxMalig_ independently for each of ADC (no filtering), ADC-M (mass/ non-mass filtering), and ADC-MD (mass/ non-mass plus SDAC directional filtering) [6]. For each of the filtering methods, ADC, ADC-M and ADC-MD, the set of potentially avoidable biopsies is the set of lesions with values above the associated ADC_MaxMalig_. By the definition of the thresholds all potentially avoidable biopsies are labeled as benign.

#### LM evaluation

The LM method allows for a non-zero fraction of malignant lesions to be downgraded to BI-RADS category 3. Negative likelihood ratio (LR-) is a measure of performance of this metric, with LR- ≤ 0.1 being a strong indication of benignity. [8] This metric requires designation of an ADC threshold with lesions having ADC values below this threshold labeled as cancer and lesions having ADC values above this threshold labeled as benign. LR- values are generated using ADC threshold equal to 1.5 × 10^-^^3^ mm^2^/s.

### Example illustrating the methodology

**Fig. 1:**
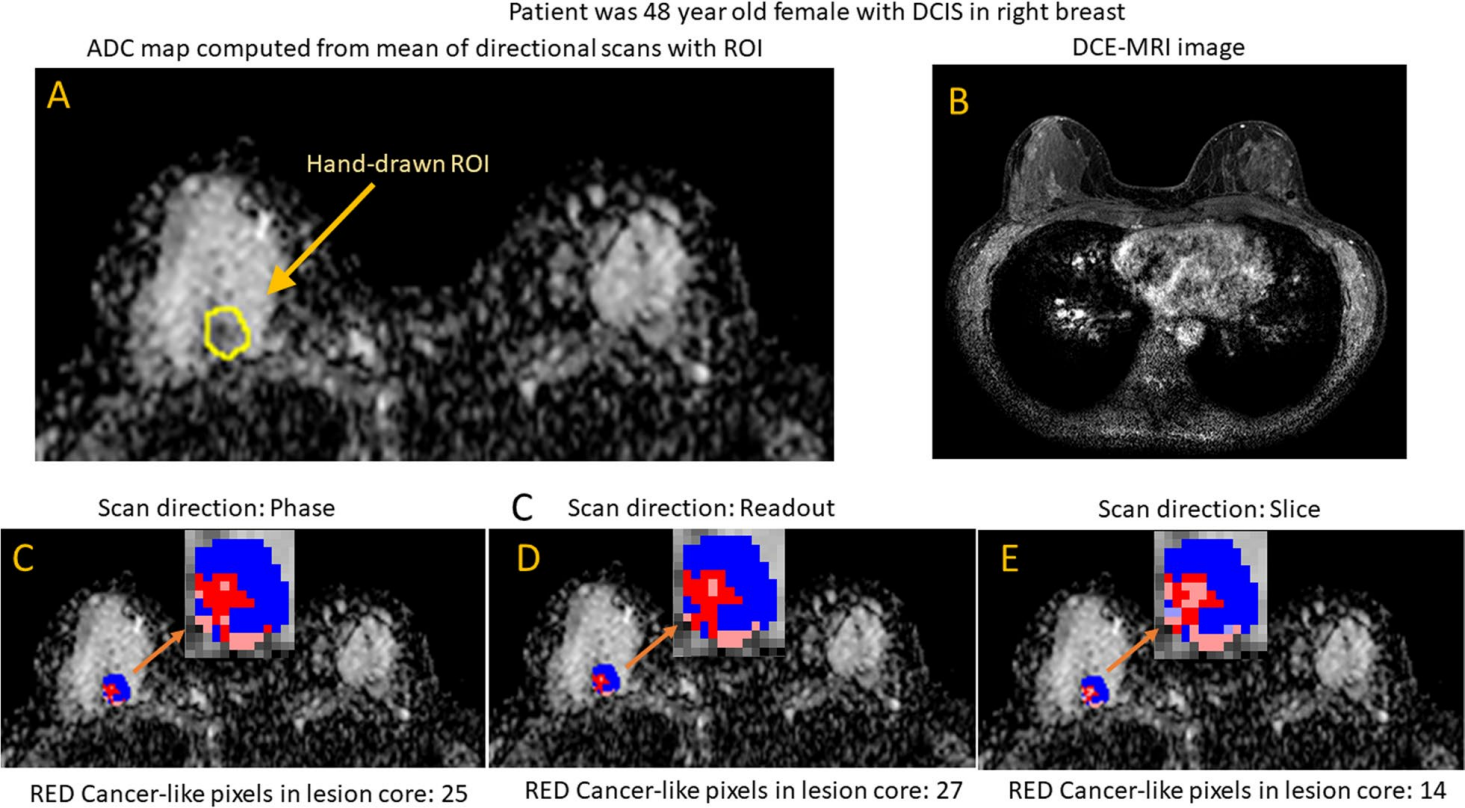
Example of SDAC filter

Figure 1 shows a 1.14 cm^2^ DCIS mass in extremely dense breasts in a 48 year old female. Figure 1A is the ADC map with hand-drawn ROI marked in yellow. Pixels are square, 1.25 mm × 1.25 mm. DWI was acquired on a 3.0 T system with repetition time (TR) = 12,976 ms, echo time (TE) = 63.89 ms, slice thickness = 2.5 mm. Figure 1B is a post-contrast DCE image at approximately the same slice location as the ADC map. Figures 1C–E show the directional ADC maps computed from scans in the phase, readout, and slice directions, with insets showing ROI enlargements.

All pixels in the ROI are colored with the following key:

**Table:** 

Hue	Intensity
Blue: Pixel ADC value ≥ 1.37 × 10^–3^ mm^2^/s (Benign-like)	Bright Colors: In lesion core
Red: Pixel ADC value < 1.37 × 10^–3^ mm^2^/s (Cancer-like)	Dull colors: Not in lesion core

Since pixel ADC values are computed as an average of values obtained on directional scans, pixel ADC values are the same for images 1C–1E, resulting in red or blue hue being the same for all three directional scans. Lesion core and associated ADC values are computed independently for each direction, resulting in intensity varying from one directional map to another (e.g., bright red in one direction may correspond to dull red in another direction).

The ROI contains 117 pixels of which 32.5% are red, denoting cancer-like ADC values (ADC < 1.37 × 10^–3^ mm^2^/s), and 67.5% are blue, denoting benign-like ADC values (ADC ≥ 1.37 × 10^–3^ mm^2^/s). The mean ADC value over the ROI is 1.534 × 10^–3^ mm^2^/s, suggesting benignity. The ADC value exceeds the 1.5 × 10^–3^ mm^2^/s threshold used in Clauser, et al. suggesting that this lesion could possibly have BI-RADS category lowered to 3 if categorization is based strictly on ADC thresholding.

SDAC evaluates variability in the surface area covered by cancer-like pixels in the lesion core, shown as bright red in the three directional maps. For each of the three directional scans, the area of cancer-like pixels is independently computed as the number of bright red pixels times the area of each pixel. The standard deviation of area covered (SDAC) is used as the measure of directional consistency, or anisotropy. The areas of bright red for the three directions are:

Direction P:39.06 mm^2^

Direction R:42.19 mm^2^

Direction S:26.56 mm^2^

The SDAC = 8.27 mm^2^ which exceed the 4.5 mm^2^ threshold, indicating increased lesion anisotropy. The SDAC filter forces this case to be excluded from automatic downgrading from Category 4 to Category 3.

### Statistical methods

Malignant lesions were considered as positive and benign lesions as negative cases in a binary classification. Improvement in specificity at 100% sensitivity for study cases was evaluated with exact McNemar test using exact2 × 2 package in R (https://CRAN.R-project.org/package=exact2x2) with 1-sided p-value < 0.05 indicating statistical significance. The negative likelihood ratio, (LR-) is defined as false-negative rate (1-sensitivity) divided by the true-negative rate (specificity). The following equations define Prob_Posttest_ relative to LR- where Prob_Pretest_ is the pre-test probability, defined as fraction of cases that are positive:

1 $${\text{Odds}}_{{{\text{Pretest}}}} = {\text{ Prob}}_{{{\text{Pretest}}}} / \, \left( {1 - {\text{Prob}}_{{{\text{Pretest}}}} } \right)$$

2 $${\text{Odds}}_{{{\text{Posttest}}}} = {\text{ Odds}}_{{{\text{Pretest}}}} *{\text{ LR - }}$$

3 $${\text{Prob}}_{{{\text{Posttest}}}} = {\text{ Odds}}_{{{\text{Posttest}}}} / \, \left( {1 + {\text{ Odds}}_{{{\text{Posttest}}}} } \right)$$

ROC graphs were constructed as plots of operating points on the (1-specificity) vs sensitivity axes. ROC graphs, constructed for ADC, ADC-M, and ADC-MD, present context for the specificity at the operating point corresponding to 100% sensitivity. The high number of lesions with assigned zero ADC values precluded meaningful area-under-curve (AUC) analysis of the ROC graph.

## Results

**Fig. 2:**
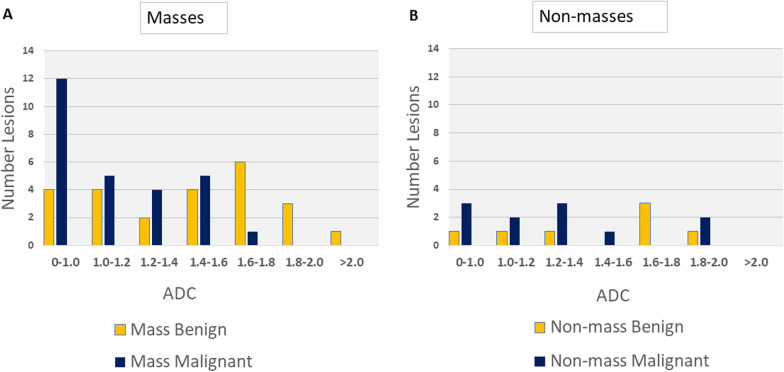
Histograms of Mass and Non-mass Lesions by ADC (10^–3^ mm^2^/s)

The non-mass designation was inclusive of, but more extensive than, NME. Both non-enhancing lesions noted in the Methods section were designated as non-mass lesions, resulting in 51 masses (24 benign; 27 malignant), and 18 non-mass lesions (7 benign; 11 malignant). Figure 2 shows ADC values of mass (2A) and non-mass lesions (2B).

**Fig. 3:**
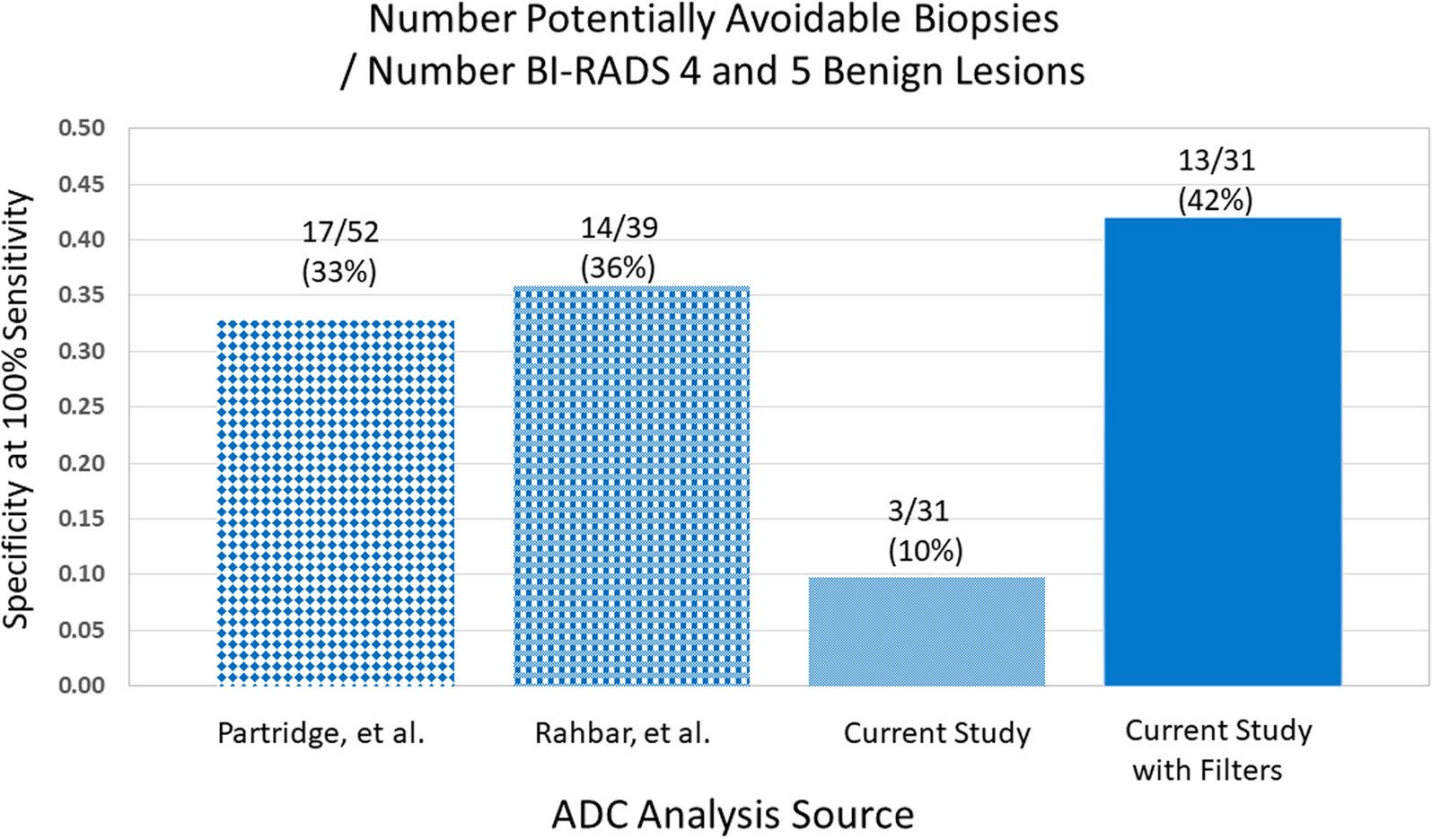
Potentially Avoidable Biopsies

For the 100% sensitivity method, our baseline analysis on our data set, computed using only ADC thresholding, showed that 3 of 31 (10%) benign lesions had potentially avoidable unnecessary biopsies. This compares with 17 of 52 (33%) reported in Partridge et al. [4], and 14 of 39 BI-RADS 4 and 5 (36%) reported in Rahbar et al. [6]. The low number of potentially avoidable unnecessary biopsies obtained from using only ADC thresholding performance on our data set was a consequence of a high maximum ADC value of 1.89 × 10^–3^ mm^2^/s for malignant lesions in our data set. The application of non-mass and directional variance filters, which resulted in some malignant lesions having their ADC-MD values forced to 0, lowered the effective maximum ADC value to 1.53 × 10^–3^ mm^2^/s. This reduction in effective maximum ADC of malignant lesions increased the number of potentially avoidable biopsies in our data set to 13 of 31 (42%). These results are shown graphically in Fig. 3.

**Fig. 4:**
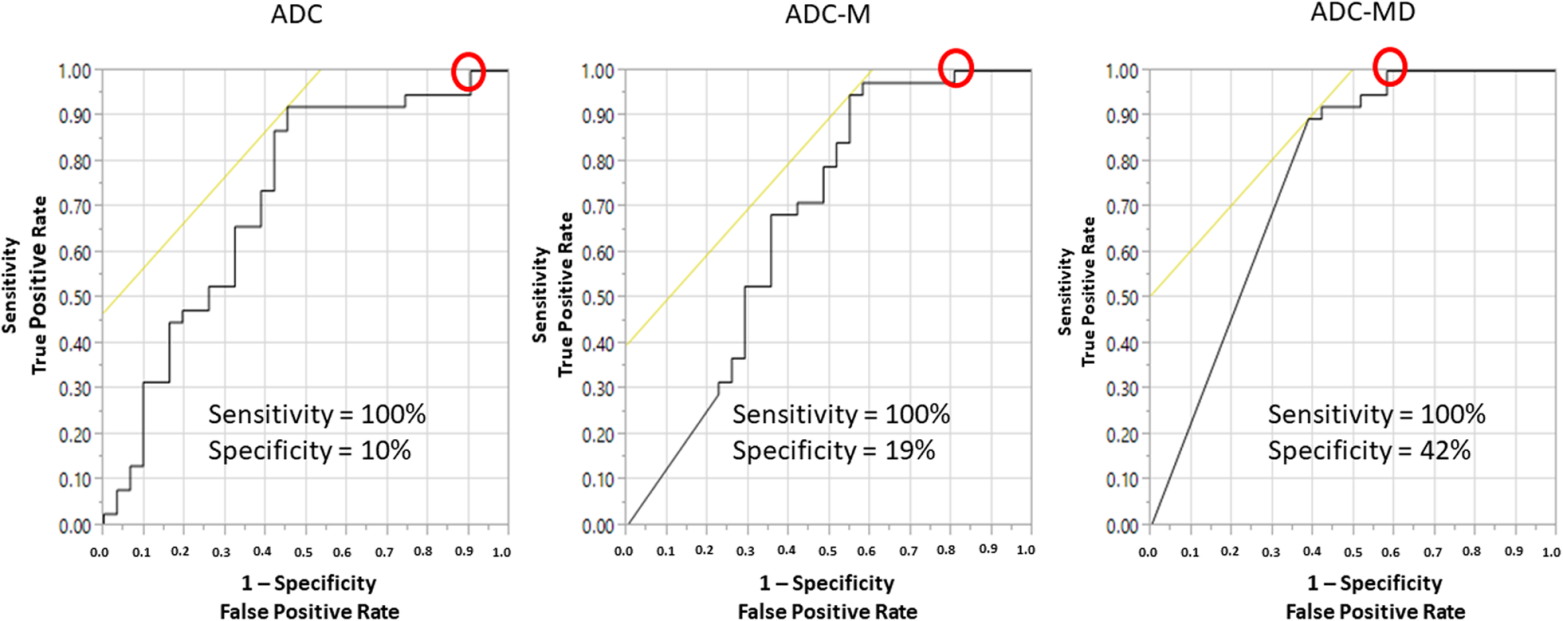
ROC curves for ADC, ADC-M, ADC-MD

Improvement from only ADC thresholding was achieved by sequential application of a mass/no-mass filter followed by a directionality filter. The 3 of 31 (10%) potentially avoidable unnecessary biopsies obtained from using only ADC thresholding (“ADC”) was increased to 6 of 31 (19%) with the application of the mass/no-mass filter (“ADC-M”), and further increased to 13 of 31 (42%) with the subsequent application of the directionality filter (“ADC-MD”). The numbers of potentially avoidable unnecessary biopsies correspond to the specificities at 100% sensitivity operating points of the ROC graphs which are marked with small circles on Fig. 4. The ADC thresholds used to achieve 100% sensitivity for ADC only and the subsequent sequential application of filters were: for ADC only, 1.89 × 10^–3^ mm^2^/s; for ADC-M, 1.73 × 10^−3^ mm^2^/s; for ADC-MD, 1.53 × 10^–3^ mm^2^/s. The improvement in specificity at 100% sensitivity on the current data for ADC-MD over specificity at 100% sensitivity for ADC alone was statistically significant (42% vs. 10%, *p* < 0.01).

For the LM method, our baseline analysis computed for ADC cutoff of 1.5 × 10^–3^ mm^2^/s on our data set, resulted in LR- = 0.27, which is an inferior result compared to LR- = 0.10 reported in Clauser, et al. [3] With the application of non-mass and directional variance filters, LR- on our data set improved to the more favorable LR- = 0.06, superior to the Clauser et al. value. [3].

Results of analysis of our data set are given along with results from comparative studies and institution and data-set statistics in Table 3 with the current study shown in bold.

**Table 3:** Dataset and ADC statistics

	100% Sensitivity Method	LM Method		ADC Thresholding only	ADC Thresholding with filters
Primary author of study	Partridge, et al	Rahbar, et al	Clauser, et al	Current Study	
Number Institutions	1	10	7	**1**	**1**
Data sets analyzed	1	1	7	**1**	**1**
Benign lesions	52	39	282	**31**	**31**
Malignant lesions	31	28	414	**38**	**38**
% Non-mass lesions	41%	42%	26%	**26%**	**26%**
Prevalence	0.373	0.346	0.595	**0.551**	**0.551**
ADC_MaxMalig_	1.81	1.53		**1.89**	**1.53**
Potentially avoidable unnecessary biopsies at ADC_MaxMalig_	17(33%)	14(36%)		**3(10%)**	**13(42%)**
LR- (ADC cutoff 1.5)			.10	**.27**	**.06**

“ADC”—apparent diffusion coefficient, “ADC_MaxMalig_”—maximum ADC value of malignant lesions, “LR-”—negative likelihood ratio, “Prevalence”—number of malignant lesions over total number of lesions

## Discussion

Our data were acquired for a prior prospective study of women with dense breasts and includes multiple malignancies with high ADC values [9] which provides a useful set of cases for studying a clinical situation. The Partridge et al. Rahbar et al. and Clauser et al. studies cited above [3, 4, 6] had clinical objectives and showed a range of values of study parameters and ADC discrimination performance measures that were comparable to those of our study and results of those studies were used for comparative analysis.

We investigated the improvements in performance with application of two new filters in addition to ADC thresholding when identifying benign lesions that can safely avoid biopsy. The effectiveness of adding DWI to DCE in reducing false positives can be adversely affected by using only ADC thresholding when the data set contains malignant lesions with high ADC values, and this reduction in effectiveness can be mitigated by using the two proposed filters.

The two proposed filters are based on mass/non-mass morphology and on directional variation in DWI signal at the lesion. MRI-DCE images, an example of which is Fig. 1B, were available to the radiologist when drawing ROIs and assessing whether a lesion was mass or NME, but the directional variation feature is computed strictly from diffusion data.

Improvement in performance is measured by comparing results obtained on our data set using ADC thresholding without filters to results obtained using ADC thresholding with filters. Performance is evaluated using two metrics that have been used in prior published studies. We then compared our results to results of these prior studies.

The first metric is the percentage of unnecessary biopsies of benign lesions that can be potentially avoided while maintaining 100% sensitivity**.** The prior studies showed potential savings of unnecessary biopsies to be 33% and 36%. Our data, using ADC thresholding without filters, showed potential savings of unnecessary biopsies of 10%. When our data were analyzed using ADC thresholding with filters the percentage savings of unnecessary biopsies improved to 42%.

The second metric used a threshold of 1.5 × 10^–3^ mm^2^/s, matching that used in the study to which we compared our results, which in turn was based on earlier published studies. 657 patients with 696 lesions from the prior multicentric study used for comparison showed LR- = 0.1 [3], just attaining the threshold. Our data, using ADC without filters showed LR- = 0.27. When our data were analyzed with ADC with filters, the results were improved to LR- = 0.06.

Our data show that malignant non-mass lesions can exhibit high ADC values which forces an unacceptably high threshold to achieve 100% sensitivity. High ADC values associated with non-masses are not unique to our dataset and have been reported previously in DCE-MRI studies. Avendano, et al., found that AUC of whole tumor ADC for NME lesions ranged from 0.53 to 0.67 for two readings from two readers, with the highest mean ADC values of: 2.67, 2.02, 1.79, and 1.63 × 10^–3^ mm^2^/s [11], and Kul, et al., found that 2 of 6 malignant NME lesions were mischaracterized using an optimum threshold that corresponded to 91.5% sensitivity [12]. The A6702 multicenter study that we used as a basis for comparison had a dataset that included 28 NME lesions, none of which were malignant with high ADC values. In contrast, our data set which included 2 malignant non-mass lesions with ADC > 1.88 × 10^–3^ mm^2^/s.

The directional variation in DWI filter was based on the standard deviation of area covered (SDAC) by pixels in the lesion core with ADC < 1.37 × 10^–3^ mm^2^/s, evaluated over the three directional DWI scans [9]. The lesion core is the intersection of the radiologist-drawn ROI and a computer-generated model constructed on augmented ADC maps. The SDAC feature introduces two new elements into the discrimination of malignant lesions. Firstly, whereas ADC is related to the number of cancer-like pixels in the ROI, SDAC is a measurement of the variability of distribution of pixels with ADC < 1.37 × 10^–3^ mm^2^/s across the three directions of the DWI acquisition. Secondly, where ADC is a measurement of the whole lesion, SDAC is computed on a core sub-ROI of the lesion. Lesion cores, or “hotspots,” have been found to be important discriminators of benign from malignant conditions [13, 14]. In order to improve the accuracy of the SDAC feature on lesion cores that have a small number of pixels with ADC < 1.37 × 10^–3^ mm^2^/s, the number of pixels being analyzed is increased by enlarging the original ROIs with the dilation operator.

The SDAC directional variance feature used in this study differs from fractional anisotropy (FA) that has been used to characterize lesion anisotropy in other studies. The SDAC feature is computed from three directional scans of DWI rather than a minimum of 6 directional scans from diffusion tensor imaging (DTI) required for FA. Several researchers have investigated FA derived from DTI as a means of quantifying anisotropy to discriminate benign from malignant breast lesions, with mixed results. Jiang, et al. [15] and Baltzer, et al., [16] found that FA was significantly higher in malignant lesions than in benign lesions, in accordance with our findings of SDAC derived from DWI. Cakir, et al. [17] and Partridge, et al. [18] found that there was no significant difference in FA between malignant and benign breast lesions.

Because SDAC only uses three directions, a true anisotropy measurement of each pixel is not possible. The SDAC feature is dependent on the orientation of the lesion relative to the scan directions. In spite of the 3-direction scan limitation, SDAC provides discriminatory effectiveness for a subset of the lesions and was shown to generate statistically significant overall discrimination between benign and malignant lesions in our data set [9]. Additional research is required to determine if similar results will be found on other data sets and if a different orientation of the three scans will improve discrimination when used for scanning for a class of breast lesions that have a predominant orientation. In addition, SDAC is computed over a lesion core whereas FA is, in general, computed over the full lesion.

When supplemental filters are used in evaluating our dataset, ADC_MaxMalig_ is reduced from 1.89 × 10^–3^ mm^2^/s to 1.53 × 10^–3^ mm^2^/s, with 10 additional true negatives (TN) included because of the reduced threshold; none of the 3 baseline TNs were affected. 4 of 10 additional TNs with ADC values between 1.53 × 10^–3^ mm^2^/s and 1.89 × 10^–3^ mm^2^/s were non-masses. The small number of non-masses suggests that the directional variance feature was an important contributor to the improvement.

Key statistics of our data compared favorably to those of the prior studies, suggesting that our data set may be representative of those found in a clinical setting. Key statistics from our study aligned with corresponding statistics from different prior studies: for example, we closely matched one prior study on number of benign lesions, a second prior study on ADC_MaxMalig_, and the third prior study on prevalence, while differing on other study pairings. Prevalence is the number of malignant lesions over the total number of lesions. [3] Comparing our study to the other two prior studies showed a dissimilar percentage of non-mass lesions and prevalence, with a similar number of malignant lesions. The inferior results we achieved using standard ADC methodology suggests that our data set may have been inherently difficult for the task of reducing the number of unnecessary biopsies of benign lesions. However, when the ADC values were modified with the two filters, our results surpassed those of all three prior studies.

We selected the ADC threshold to be 1.50 × 10^–3^ mm^2^/s, following the method of Clauser, et al. who selected that value by rounding down ADC_MaxMalig_ = 1.53 × 10^–3^ mm^2^/s obtained by Rahbar in the A6702 analysis.[3, 6] Clauser, et al. noted: “Our study confirms this ADC cutoff in a considerably large, multicentric dataset across independent centers, MRI vendors, and readers." [3] If we had used the unrounded 1.53 × 10^–3^ mm^2^/s value reported by Rahbar, rather than the 1.5 × 10^–3^ mm^2^/s roundoff, the threshold would have coincidentally matched our ADC_MaxMalig_, giving the result of 0 FNs, the same as achieved using the 100% sensitivity method. By using a 1.50 × 10^–3^ mm^2^/s cutoff, we followed the methodology of Clauser, et al. and were able to evaluate different results using the two methods on our data.

The methodology used here and in the trial is based on a single high ADC threshold to bifurcate the data set into lesions with sufficiently high ADC values to be labeled as benign and other lesions which includes all of the malignancies. Zhang, et al., used a low ADC threshold (1.00 × 10^−3^ mm^2^/s) to identify lesions with sufficiently low ADC values to be labeled as cancer [19]. For the cases used in both this study and A6702, a low lesion threshold would have resulted in excessive FPs and was not used. Additional case data and research is needed to investigate whether a low ADC threshold may be useful for discrimination in a different patient population; for example, in a non-contrast screening rather than a diagnostic paradigm.

## Limitations

A limitation of our study is that there are only 38 malignant and 31 benign lesions. A consequence of this limitation is that FNs were evaluated relative to a threshold at 2% where each of the malignant lesions represented 2.6% (1/38) of the total number. We believe, however, that the results of our study, the relative insensitivity of the thresholds to minor variation, and a patient population that reflects community settings provide evidence for potential clinical utility of the proposed methodology. A related limitation is the inclusion of two NMLs that did not enhance on DCE-MRI. For these two lesions, the ADC measurements were done with reference to mammography and/or ultrasound, rather than DCE-MRI, which could introduce errors.

A third limitation of the study is that the cases included 5 lesions imaged at 1.5 T and 64 lesions at 3.0 T. We elected to include the small number of cases imaged at 1.5 T to preserve the prospective nature of the study; the use of both 1.5 T and 3.0 T systems is consistent with the A6702 study. The three malignant lesions imaged at 1.5 T had ADC values equal to 0.908, 0.906, and 1.449 × 10^−3^ mm^2^/s which were below the maximum ADC value of lesions imaged at 1.5 T and the 1.5 × 10^−3^ mm^2^/s threshold used for LM evaluation and had minimal effect on overall results.

A fourth limitation is restricting the study to dense breasts; additional research is needed to determine if these results generalize to a larger population. A fifth limitation is that the study was conducted at a single institution with ROIs drawn by a single reader. A sixth limitation is that we evaluated our results relative to those of only three prior studies: a single institution study, a multi-institution study, and a meta-analysis of data from 7 studies. The improvements resulting from implementation of the non-mass and SDAC filters were promising, but with the small number of cases and the large number of lesions assigned the value 0 by the ADC-plus-filters, the results need to be validated in larger multi-center follow-up studies.

## Conclusion

In this analysis based on prospective data in a cohort of women with dense breasts, we demonstrated that sequential application of lesion type-based (mass vs non-mass) and directional-variability-based filters in addition to ADC thresholding resulted in statistically significant improvement in the number of avoidable unnecessary biopsies while maintaining 100% sensitivity. We also showed that use of the filters substantially reduced LR- to levels that met criteria which allow for a minimal number of malignancies to be classified as BI-RADS Category 3.

## Data Availability

The data sets analyzed during the current study are not publicly available due to limitations of the IRB but are available from the corresponding author upon approval of the P.I. of the IRB.
